# ^18^F-FDG-PET Detects Drastic Changes in Brain Metabolism in the Tg4–42 Model of Alzheimer’s Disease

**DOI:** 10.3389/fnagi.2018.00425

**Published:** 2019-01-08

**Authors:** Caroline Bouter, Philipp Henniges, Timon N. Franke, Caroline Irwin, Carsten Oliver Sahlmann, Marius E. Sichler, Nicola Beindorff, Thomas A. Bayer, Yvonne Bouter

**Affiliations:** ^1^Department of Nuclear Medicine, University Medical Center Göttingen (UMG), Georg-August-University, Göttingen, Germany; ^2^Division of Molecular Psychiatry, Department of Psychiatry and Psychotherapy, University Medical Center Göttingen (UMG), Georg-August-University, Göttingen, Germany; ^3^Berlin Experimental Radionuclide Imaging Center (BERIC), Charité—University Medicine Berlin, Berlin, Germany

**Keywords:** positron-emission tomography, ^18^F-Fluorodeoxyglucose, brain metabolism, Alzheimer’s disease, N-truncated Aβ, neuron loss, behavior, transgenic mouse models

## Abstract

The evaluation of new therapeutic strategies in Alzheimer’s disease (AD) relies heavily on *in vivo* imaging and suitable animal models that mimic the pathological changes seen in patients. ^18^F-Fluorodeoxyglucose (^18^F-FDG)-positron-emission tomography (PET) is a well-established non-invasive imaging tool for monitoring changes in cerebral brain glucose metabolism *in vivo*. ^18^F-FDG-PET is used as a functional biomarker for AD as patients show an early and progressive reduction of cerebral glucose metabolism. However, earlier studies in preclinical models of AD showed conflicting results. The aim of this study was the evaluation of cerebral glucose metabolism in the Tg4–42 mouse model of AD using ^18^F-FDG-PET/magnetic resonance imaging (MRI). Tg4–42 mice show an age-dependent reduction in glucose metabolism together with severe neuron loss and memory deficits. Similar to AD patients early decrease in ^18^F-FDG uptake was already detected in young (3 months) Tg4–42 mice. The altered glucose metabolism coupled with age- and disease related cognitive decline of Tg4–42 mice make it a well-suited model for preclinical testing of AD-relevant therapeutics.

## Introduction

Alzheimer’s disease (AD) is the most common form of dementia accounting for 60%–80% of all cases. The neurodegenerative disease is characterized by behavior and cognitive symptoms due to functional and structural abnormalities including accumulation of amyloid-β (Aβ) protein, neurofibrillary tangles and neuronal loss (Holtzman et al., 2011).

According to the amyloid cascade hypothesis the deposition of Aβ is the causative event of AD pathology (Hardy and Allsop, 1991). Aβ is generated through sequential cleavage of the amyloid precursor protein (APP) by β- and γ-secretases (Selkoe, 1998; Zhang et al., 2011). Next to Aβ peptides starting with an aspartate as the first amino acid, a variety of different *N*-truncated Aβ peptides have been identified in AD brains. N-terminal deletion enhances neurotoxicity and Aβ aggregation (Pike et al., 1995; Bayer and Wirths, 2014). There is substantial evidence that N-terminal truncated peptides play a key role in AD and they are highly abundant in brains of patients diagnosed with sporadic and familial AD (Jawhar et al., 2011; Bayer and Wirths, 2014; Dunys et al., 2018). Aβ4–42 is a particular abundant Aβ species in AD and one of the major fractions in the cortex and hippocampus of AD patients (Masters et al., 1985; Portelius et al., 2010). In order to further elucidate the toxicity of Aβ4–42 the transgenic mouse model Tg4–42 was generated. This AD mouse model overexpresses Aβ4–42 without human APP overexpression and without any mutations related to familial AD (Bouter et al., 2013). N-truncated Aβ4–42 triggers age-dependent neuron loss and behavior deficits comparable to AD-typical memory dysfunction albeit without plaque formation and neurofibrillary tangles (Antonios et al., 2015). While hippocampal atrophy caused by neuron death is one of the major hallmarks of AD progression only a small fraction of AD models show this key feature of AD (Bayer and Wirths, 2010).

The development of new treatment strategies against AD relies to a great extent on the use of transgenic mouse models. The massive neuron loss coupled with age- and disease-related cognitive decline make the Tg4–42 model well suited for preclinical testing of AD-relevant therapeutics. However, a suitable mouse model for preclinical trials also requires the validation of the same *in vivo* biomarkers that can be used in patients. To date, biomarkers for AD include measurement of Aβ42, total (t-tau) and phosphorylated (p-tau) in cerebrospinal fluid as well as positron-emission tomography (PET) and magnetic resonance imaging (MRI; Jack et al., 2013, 2018). PET displays a non-invasive imaging tool for the detection of Aβ deposition with amyloid PET or measuring cerebral glucose metabolism with ^18^F-Fluorodeoxyglucose (^18^F-FDG)-PET. ^18^F-FDG-PET is able to detect neuronal dysfunction and neurodegeneration *in vivo* as brain glucose metabolism is determined by synaptic activity mainly in order to restore membrane potentials (Kadekaro et al., 1985). In neurodegenerative disorders synaptic activity and glucose metabolism can be disturbed long before neuron loss occurs. ^18^F-FDG-PET can detect those changes in order to identify cerebral areas with reduced glucose metabolism. In the clinical routine ^18^F-FDG-PET is used to differentiate between different neurodegenerative diseases as certain patterns of reduced glucose uptake are specific for various disorders. A meta-analysis of Bloudek et al. (2011) evaluating FDG-PET in the diagnosis of AD showed a sensitivity of 91% and a specificity of 86%. Patients show an early and progressive reduction of glucose metabolism in the posterior cingulum, precuneus and temporoparietal cortex and hippocampus (Mosconi, 2013). Small animal PET scanners allow measuring the same molecular processes in animal models as in humans. Ideally functional, structural and molecular changes observed in patients should also be detected in a similar way in an animal model of AD.

In this study we used *in vivo* imaging with ^18^F-FDG-PET to evaluate the glucose metabolism in Tg4–42 mice under resting conditions. The aim of the present report was to investigate if the glucose metabolism could be an early biomarker to distinguish Tg4–42 animals from wild-type (WT) mice.

## Materials and Methods

### Tg4–42 Transgenic Mice

The generation of Tg4–42 mice has been described previously (Bouter et al., 2013). Briefly, Tg4–42 mice express human Aβ4–42 fused to the murine thyrotropin-releasing hormone signal peptide, ensuring secretion through the secretory pathway, under the control of the neuronal Thy-1 promoter. Tg4–42 mice were generated and maintained on a C57Bl/6J genetic background. Only homozygous Tg4–42 mice were used in this study. If not stated otherwise an equal distribution of male and female animals was used in all experiments.

All animals were handled in accordance with the German guidelines for animal care and experiments were approved by the local authorities (Niedersächsisches Landesamt für Verbraucherschutz und Lebensmittelsicherheit, Röverskamp 5, 26203 Oldenburg, Germany and Landesamt für Gesundheit und Soziales LAGeSo Darwinstr. 15, 10589 Berlin).

### PET/MRI Image Acquisition and Analysis

Young (3–4 months) and aged (7–8 months) Tg4–42 (*n* = 7, female) and aged WT C57Bl/6J (7–8 months, *n* = 5, female) control mice were fasted overnight and blood glucose levels were measured before tracer injection. 9–21 MBq (mean 16.27 MBq) ^18^F-FDG was administered intravenously into a tail vein with a maximum volume of 200 μl and scans were performed after an uptake period of 45 min. Mice were anesthetized with isoflurane supplemented with oxygen during the scans and were awake during the uptake period. PET imaging was performed on a small animal 1 Tesla nanoScan PET/MRI (Mediso, Hungary). Mice were kept on a heated bed at 37°C during the scan. Respiration rate was monitored constantly during the scans. PET scans were performed for 20 min. MRI-based attenuation correction was conducted with the material map (matrix 144 × 144 × 163 with a voxel size of 0.5 × 0.5 × 0.6 mm^3^, TR: 15 ms, TE 2.032 ms and a flip angle of 25°) and the PET images were reconstructed with the following parameters: matrix 136 × 131 × 315 with a voxel size of 0.23 × 0.3 × 0.3 mm^3^.

**Figure 1:**
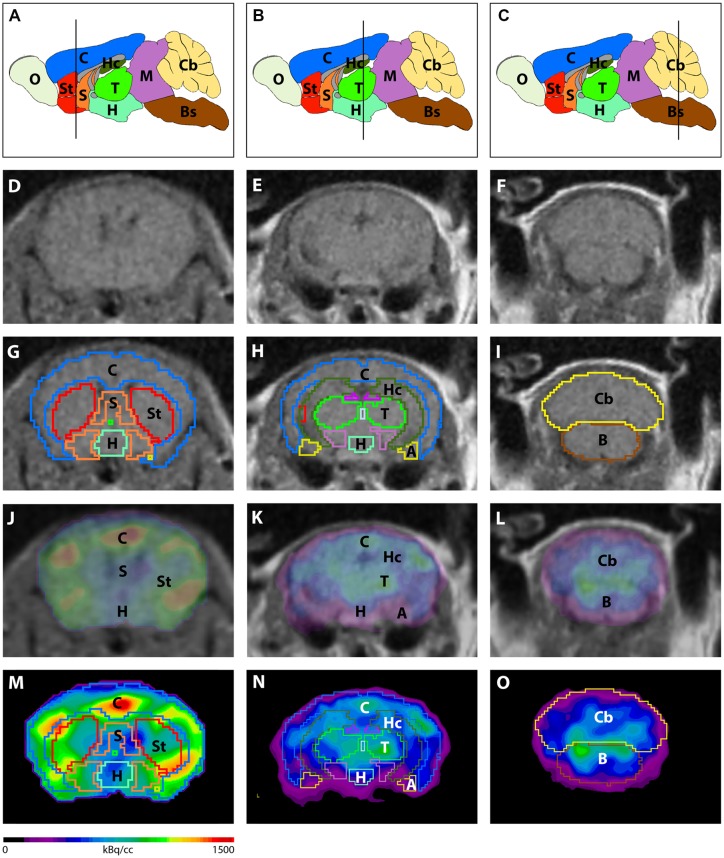
Image analysis using a mouse brain atlas. **(A–C)** Illustration of brain regions that are ascertained by the mouse brain atlas in sagittal view. Lines indicate the level of coronal slices shown in **(D–O)**. Magnetic resonance imaging (MRI) images **(D–F)** were used to match volumes of interest (VOIs) of the mouse brain atlas to individual brains of each mouse. **(G–I)** Examples of predefined VOIs matching the MRI in coronal view. Positron-emission tomography (PET) images were fused to the MRI **(J–L)** in order to match VOIs to the PET images **(M–O)**. A, Amygdala; Bs, Brain Stem; C, Cortex; Cb, Cerebellum; H, Hypothalamus; Hc, Hippocampus; M, Midbrain; O, Olfactory Bulb; S, Septum/Basal Forebrain; St, Striatum; T, Thalamus.

Image analysis was performed using PMOD v3.9 (PMOD Technologies, Switzerland). A predefined mouse brain atlas template was used to analyze different brain regions. Next to the whole brain volumes of interest (VOIs) a total of 11 predefined VOIs were matched to the mouse brain MRI (A, Amygdala; BS, Brain Stem; Cb, Cerebellum; C, Cortex; Hc, Hippocampus; H, Hypothalamus; M, Midbrain; O, Olfactory Bulb; S, Septum/Basal Forebrain; St, Striatum and T, Thalamus; Figure 1). Corresponding PET images were matched to the MRI and VOI statistics in kBq/cc were generated for all brain areas. Standardized uptake value (SUV) was calculated [SUV = tissue activity concentration average (KBq/cc) × body/weight (g)/injected dose (kBq)] for semi-quantitative analysis. Afterwards, SUV values were corrected for blood glucose levels [SUV_Glc_ = SUV × blood glucose level (mg/dl)].

### Behavior Experiments

Mice were kept on a 12 h/12 h inverted dark/light cycle and behavior experiments were performed during the dark phase.

Spatial reference memory in Tg4–42 mice was evaluated using the Morris water maze (Morris, 1984; Vorhees and Williams, 2006). Young (3 months) and aged (7–8 months) Tg4–42 and WT C57Bl/6J mice were tested in the water maze (*n* = 14–15) as described previously (Bouter et al., 2014). In brief, mice learn to use spatial cues to locate a hidden platform in a circular pool filled with opaque water. The probe trial was used to assess spatial reference memory. The platform was removed from the pool, and mice were introduced into the water from a novel entry point. Mice were then allowed to swim freely for 1 min while their swimming path was recorded. After the probe trial, the mice were sacrificed. ANY-Maze video tracking software (Stoelting Co., Wood Dale, IL, USA) was used to record swimming path, swimming speed, latency and quadrant preference.

Novel object recognition is a commonly used test for recognition memory in rodents (Leger et al., 2013; Cohen and Stackman, 2015; Grayson et al., 2015). The novel object recognition test was performed in a 50 × 50 cm arena made of gray plastic (Jawhar et al., 2012). Aged mice (7–8 months, *n* = 12–14) were allowed to familiarize with the area for 5 min. Twenty-four hours after the open field test mice were presented with two identical objects in the arena. Mice were allowed to freely explore the apparatus for 5 min. Twenty-four hours later, mice were placed back in the arena (5 min) that contained the familiar and a novel object. Because mice have an innate preference for novelty, mice that recognize the familiar object will spend most of their time with this object (Lueptow, 2017).

ANY-Maze video tracking software was used to record the time mice spent with each object and the distance traveled. The objects and the arena were cleaned with alcohol between each mouse to remove any lingering scents.

### Quantification of Neuron Numbers Using Unbiased Stereology

Unbiased stereology was used to quantify the number of neurons in different brain regions as previously described (Bouter et al., 2013). Therefore, 7-month-old mice were anesthetized and transcardially perfused with 4% paraformaldehyde. Left brain hemispheres were fixed in 4% paraformaldehyde, cryoprotected in 30% sucrose, frozen and frontally cut into a series of 30-μm thick sections on a cryostat (Microm HM550, Germany). Every tenth section was systematically sampled and stained with cresyl violet. Stereological analysis of the hippocampal cell layer CA1 (Bregma −1.22 to −3.80 mm), the hippocampal cell layer CA2/CA3 (Bregma −0.94 to −3.52 mm) and the dentate gyrus (DG; Bregma −1.34 to −3.80 mm) were performed in aged Tg4–42 and WT mice as previously described using a stereology workstation [Olympus BX51 with a motorized specimen stage for automatic sampling, StereoInvestigator 7 (MicroBrightField, Williston, VT, USA)]. The volume of the CA1, CA2/CA3 and DG were estimated by using Cavalieri’s principle (Rosen and Harry, 1990).

### Immunohistochemistry on Paraffin Brain Sections

Mice were transcardially perfused with 4% paraformaldehyde in phosphate-buffered saline. Brains were then carefully dissected, postfixed in 4% buffered formalin and paraffin embedded. Immunohistochemistry on 4 μm sagittal paraffin sections was performed as described previously (Bouter et al., 2015). The Aβ42 antibody (1:500, rabbit, Synaptic Systems, Göttingen), IBA-1 (1:1,000, guinea pig, Synaptic Systems) and GFAP (1:1,000, rabbit, Synaptic Systems) were used. Biotinylated secondary anti-rabbit and anti-guinea pig antibodies (1:200) were purchased from Jackson (ImmunoResearch Laboratories, West Grove, PA, USA). Staining was visualized using the ABC method, with a Vectastain kit (Vector Laboratories, Burlingame, CA, USA) and diaminobenzidine as chromogen. Counterstaining was carried out with hematoxylin.

Microgliosis and astrogliosis were evaluated in the hippocampal area using an Olympus BX51 microscope equipped with a MoticamPro 282B digital camera. For each antibody (IBA-1; GFAP) three images (DG, CA1, CA2/3) with 20× magnification were captured on three sections per mouse 30 μm apart from each other. The microscope- and exposure-settings remained constant during the analysis of each marker. Using ImageJ (V 1.51, NIH, Bethesda, MA, USA) the pictures were binarized to 8-bit black and white images and a fixed intensity threshold was applied defining the DAB signal. The percentage area covered by positive DAB staining was measured for each image (Jawhar et al., 2012).

### Statistical Analysis

Differences between groups were tested with unpaired *t-*tests or one-way analysis of variance (ANOVA) followed by Bonferroni multiple comparison as indicated. All data are given as mean ± standard error of the mean (SEM). Significance levels are given as follows: ***p < 0.001; **p < 0.01; *p < 0.05. GraphPad Prism version 6.07 for Windows (GraphPad Software, San Diego, CA, USA) was used for all calculations.

## Results

### Decreased Cerebral Glucose Metabolism in Tg4–42 Mice

^18^F-FDG-PET was used to evaluate brain glucose metabolism in young and aged Tg4–42 and WT mice. For quantitative analysis ^18^F-FDG uptake was measured in different brain areas using predefined VOIs and blood glucose corrected SUV values (SUV_Glc_) were calculated. ^18^F-FDG uptake was visible in all cerebral areas and the cerebellum in all tested mice. All mice showed a physiological extracranial ^18^F-FDG distribution with normal uptake in the Harderian glands, brown adipose tissue, myocardium, kidneys, gastro-intestinal tract and urinary bladder.

**Figure 2:**
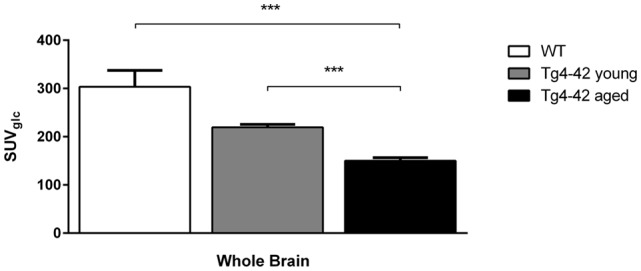
^18^F-Fluorodeoxyglucose (^18^F-FDG) uptake in the whole brain. Aged Tg4–42 mice showed a significantly lower SUV_Glc_ in the whole brain compared to wild-type (WT) and young Tg4–42 mice. WT and young Tg4–42 mice did not show significant differences in SUV_Glc_ in the whole brain. Unpaired *t*-test; ***p < 0.001; data presented as mean ± SEM.

**Figure 3:**
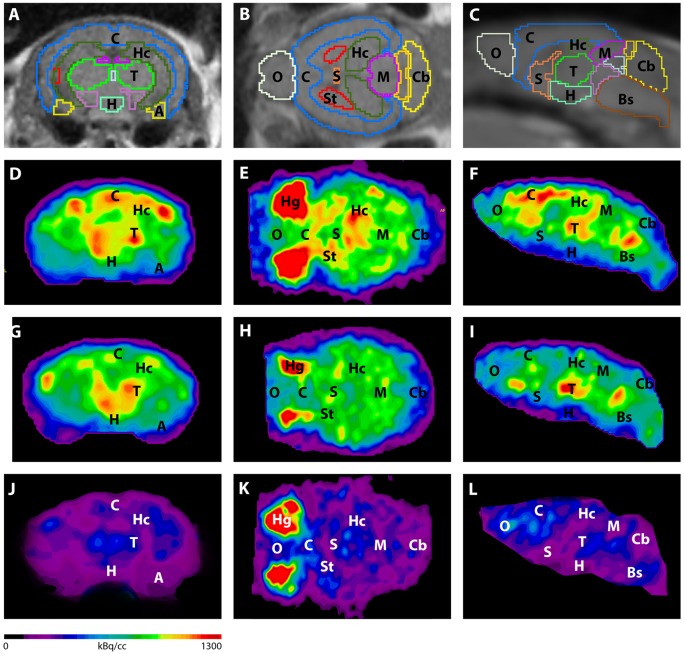
Imaging of ^18^F-FDG uptake in WT mice compared to young and aged Tg4–42 mice. **(A–C)** MRI images were matched with predefined brain regions; coronal, transverse and sagittal view. **(D–F)** ^18^F-FDG-PET images of a WT mouse in coronal, transverse and sagittal view. **(G–I)** ^18^F-FDG-PET images of a young Tg4–42 mouse in coronal, transverse and sagittal view. **(J–L)** ^18^F-FDG-PET images of an aged Tg4–42 mouse in coronal, transverse and sagittal view. ^18^F-FDG uptake was distinctly lower in aged Tg4–42 mice compared to WT mice. In young Tg4–42 mice ^18^F-FDG uptake did not show significant differences in whole brain uptake but was reduced in the hippocampus, forebrain, hypothalamus, amygdala and midbrain. A, Amygdala; Bs, Brain Stem; C, Cortex; Cb, Cerebellum; H, Hypothalamus; Hc, Hippocampus; Hg, Harderian glands; M, Midbrain; O, Olfactory Bulb; S, Septum/Basal Forebrain; St, Striatum; T, Thalamus.

Aged Tg4–42 mice had a significantly lower SUV_Glc_ in the whole brain compared to WT and young Tg4–42 mice (*t*-test: *p* < 0.001; Figure 2). WT and young Tg4–42 mice did not show significant differences in SUV_Glc_ in the whole brain (*t*-test: *p* = 0.1135; Figure 2). Figure 3 shows exemplary ^18^F-FDG-PET results of WT mice compared to young and aged Tg4–42 mice.

**Figure 4:**
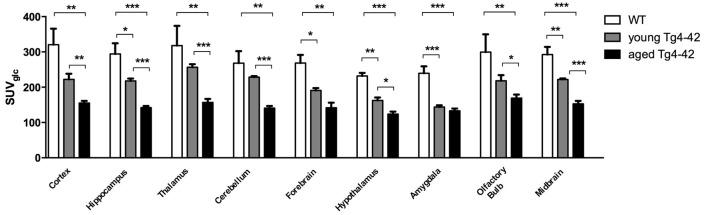
Quantification of ^18^FDG uptake in all brain areas. Aged Tg4–42 mice showed significantly lower SUV_Glc_ in all brain regions compared to WT. Differences between WT and young Tg4–42 mice were observed in the hippocampus, forebrain, hypothalamus, amygdala and midbrain. Aged Tg4–42 mice had lower SUV_Glc_ in all areas except of amygdala and forebrain compared to young Tg4–42 mice. Unpaired *t*-test; ***p < 0.001, **p < 0.01, *p < 0.05 data presented as mean ± SEM.

Differences between SUV_Glc_ of WT and old Tg4–42 mice were detected in all tested brain regions (*t*-test: cortex: *p* = 0.0015; hippocampus: *p* < 0.0001; thalamus: *p* = 0.0047; cerebellum: *p* = 0.0011; forebrain: *p* = 0.0018; hypothalamus: *p* < 0.0001; amygdala: *p* < 0.0001; olfactory bulb: *p* = 0.0086; midbrain: *p* < 0.0001; Figure 4). Compared to young Tg4–42 mice aged Tg4–42 mice had lower SUV_Glc_ in all areas except of amygdala and forebrain (*t*-test: *p* = 0.0011; hippocampus: *p* < 0.0001; thalamus: *p* = 0.0004; cerebellum: *p* < 0.0001; forebrain: *p* = 0.0583; hypothalamus: *p* < 0.0129; amygdala: *p* < 0.2876; olfactory bulb: *p* = 0.0289; midbrain: *p* < 0.0001; Figure 4). Differences between WT and young Tg4–42 mice were observed in the hippocampus, forebrain, hypothalamus, amygdala and midbrain (*t*-test: hippocampus: *p* = 0.0325; forebrain: *p* = 0.0316; hypothalamus: *p* = 0.0043; amygdala: *p* = 0.0008; midbrain: 0.0091; Figure 4). Remaining areas did not show significant differences in SUV_Glc_ between WT and young Tg4–42 mice (*t*-test: cortex: *p* = 0.1116; thalamus: *p* = 0.3416; cerebellum: *p* = 0.3065; olfactory bulb: *p* = 0.1987; Figure 4). Figure 3 shows exemplary ^18^F-FDG-PET results of WT mice compared to young and aged Tg4–42 mice.

### Neuron Loss in Hippocampus of Aged Tg4–42 Mice

**Figure 5:**
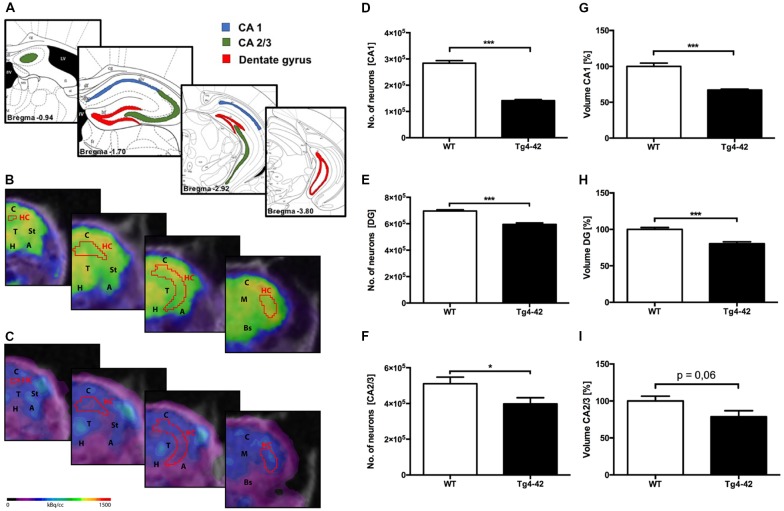
Pronounced neuron loss, hypometabolism and volume reduction in the hippocampus of aged Tg4–42 mice. **(A)** Unbiased stereology was performed to quantify the number of neurons in CA1, dentate gyrus (DG) and CA2/3 region. **(B,C)** Fused ^18^F-FDG-PET/MRI images of 7-month-old WT mice **(B)** and 7-month-old Tg4–42 mice **(C)** in coronal view. Slices are displayed according to regions in **(A)**. Hippocampus VOI highlighted in red. FDG uptake in the Hippocampus of 7 months old Tg4–42 mice was distinctly lower compared to WT. **(D,G)** Seven-month-old Tg4–42 mice displayed a significant neuron loss of ~50% and a volume reduction of ~33% in CA1 region. **(E,H)** Quantification of neurons in 7-month-old Tg4–42 mice in DG revealed a significant neuron loss of 14% and a volume reduction of 20%. **(F,I)** Aged Tg4–42 mice exhibited a significant loss of neurons in CA 2/3 region of the hippocampus but no volume reduction. Schematic illustration of the counting areas **(A)** modified from Paxinos and Franklin (2011). A, Amygdala; Bs, Brain Stem; C, Cortex; H, Hypothalamus; HC, Hippocampus; M, Midbrain; St, Striatum; T, Thalamus. Unpaired *t-*test; ***p < 0.001, *p < 0.05; data presented as mean ± SEM.

To analyze the neuron loss in Tg4–42 mice unbiased design-based stereological analysis was performed in various regions of the hippocampus (Figure 5A). Seven-month-old homozygous Tg4–42 mice exhibited a 50% neuron loss (141,568; SEM ± 3,982; *t*-test: *p* < 0.001; Figures 5C,D) and a volume reduction of 33% (*t*-test: *p* < 0.001; Figure 5G) in the CA1 layer of the hippocampus compared to WT controls (284,072; SEM ± 9,884; Figures 5B,D,G). Furthermore, aged Tg4–42 mice showed a significant loss of neurons in DG of 14% (595,016; SEM ± 11,308) compared to WT controls (695,651; SEM ± 9,029, *t*-test: *p* < 0.001; Figure 5E). Further quantitative analysis of the DG volume demonstrated a significant volume reduction of 20% in Tg4–42 mice (*p* < 0.001; Figure 5H). Aged Tg4–42 mice also display a significantly reduced neuron number in the CA2/3 region of the hippocampus (398,338; SEM ± 34,074) compared to WT control animals (510,492; SEM ± 36,819, *t*-test: *p* = 0.049; Figure 5F). The corresponding volume of CA2/3 did not differ significantly between the two groups (*t*-test: *p* = 0.06; Figure 5I).

### Impaired Spatial Reference and Recognition Memory in Aged Tg4–42 Mice

**Figure 6:**
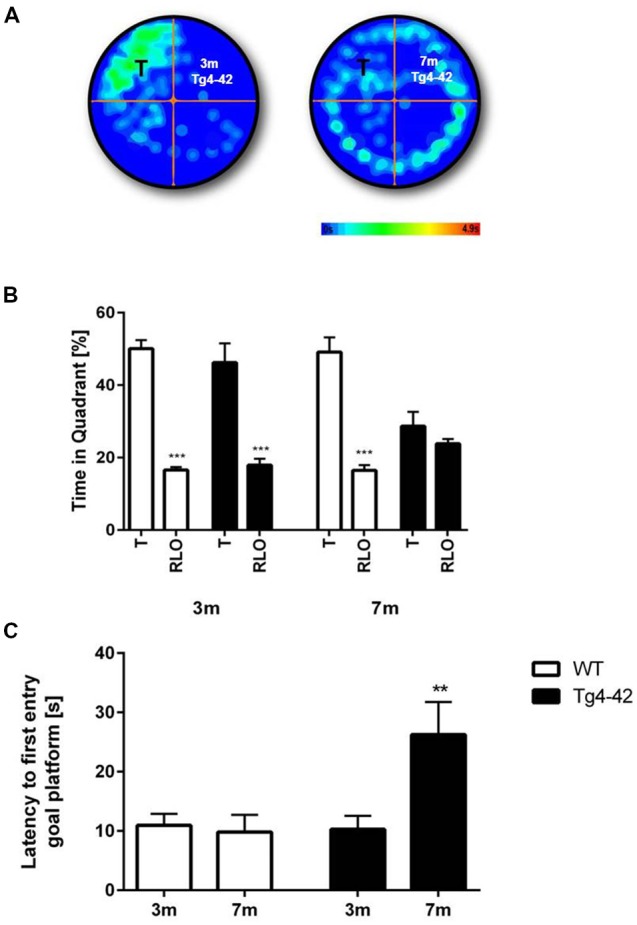
Reference memory deficits in the water maze in Tg4–42 mice.** (B)** WT mice and 3-month-old Tg4–42 mice showed an intact spatial reference memory as they spent significantly more time in the target quadrant while 7-month-old Tg4–42 had no preference for the target quadrant in the probe trial of the water maze. **(A)** This is exemplified by heat maps of 3-month-old Tg4–42 and 7-month-old Tg4–42 mice. **(C)** Aged Tg4–42 showed a significantly longer latency to reach the goal platform in the probe trial than same-aged WT mice or young Tg4–42 mice. T, target; RLO, right/left/opposite. *t*-test; ***p < 0.001; **p < 0.01; data presented as mean ± SEM; m, months.

Spatial reference memory was assessed in the probe trial of the Morris water maze. Young Tg4–42 and WT mice displayed a significant preference for the target quadrant, as indicated by the percentage time spent in the target quadrant of the pool (Figures 6A,B, *t*-test, target vs. all other quadrants: WT: *p* < 0.001; Tg4–42 *p* < 0.001). No quadrant preference was found for aged Tg4–42 mice, while WT mice still demonstrated significant preference for the target quadrant (Figure 6B, *t*-test, target vs. all other quadrants: WT: *p* < 0.001; Tg4–42 *p* = 0.2553). The absence of a preference for the target quadrant as compared to the remaining quadrants during the probe trial demonstrates that aged Tg4–42 mice display a robust deficit in spatial reference memory. Furthermore, 7-month-old Tg4–42 mice showed a significant longer latency to enter the target quadrat than same-aged WT animals (Figure 6C, *t*-test, *p* = 0.0059). Swimming speed between the groups did not differ during the probe trial (data not shown).

**Figure 7:**
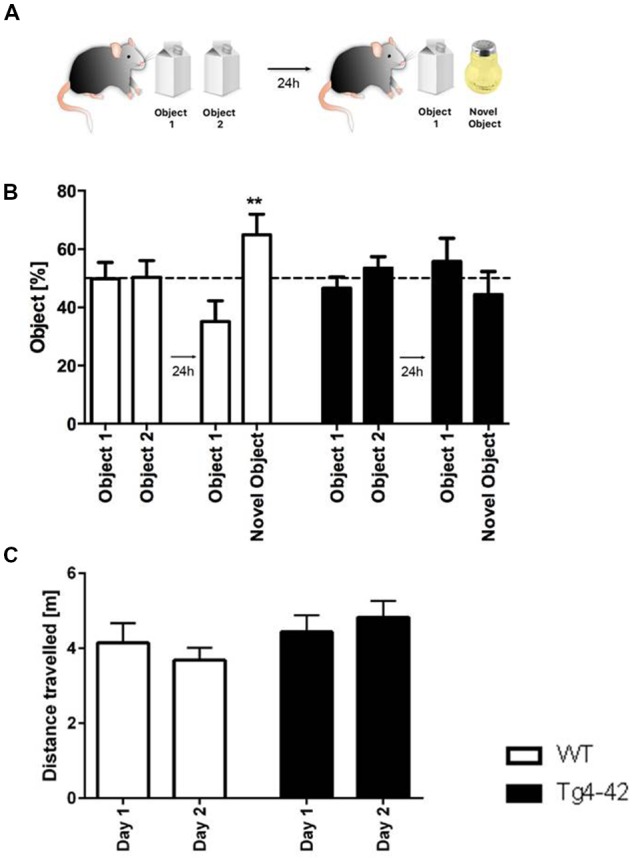
Cognitive deficits in Tg4–42 mice. **(A)** Schematic representation of the novel object recognition task. **(B)** In the exploration phase of the novel object test, 7-month-old WT and Tg4–42 mice spent ~50% of the time with both objects. During the test trial, only WT mice showed a clear preference for the novel object, whereas Tg4–42 mice showed no preference for one of the objects. **(C)** Distance traveled did not alter between WT and Tg4–42 mice in the novel object task. **(B)** One-way analysis of variance (ANOVA) followed by Bonferroni multiple comparison; **(C)** unpaired *t*-test; **p < 0.01; data presented as mean ± SEM.

In addition, recognition memory in aged Tg4–42 mice was accessed in the novel object recognition task (Figure 7A). In this assay mice must differentiate between a familiar and a novel object and the test is based on the spontaneous tendency of mice to spend more time exploring a novel object. On the first day Tg4–42 and WT animals explored the identical objects equally. When tested for recognition memory the following day only WT animals were able to discriminate between the novel and the familiar object (Figure 7B, one-way ANOVA *p* < 0.01). In contrast, Tg4–42 mice did not show a preference for any of the objects (Figure 7B, one-way ANOVA *p* = 0.5418). Distance traveled did not differ between WT and Tg4-42 mice (Figure 7C).

### Aβ4–42-Expression and Gliosis in Tg4–42 Mice

**Figure 8:**
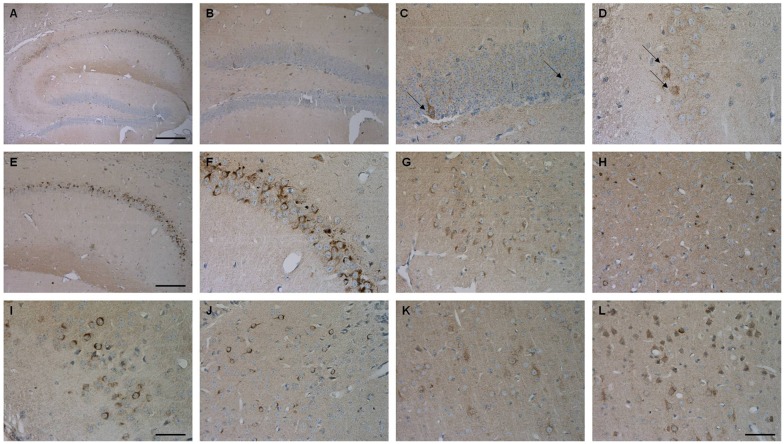
Aβ4–42 expression in various brain regions in Tg4–42 mice. Tg4–42 brain sections showed strong Aβ immunoreactivity in the brain predominantly in the CA1 region of the hippocampus **(A,E,F)**. Aβ positive cells in the DG **(B,C)** and CA3 **(D)** region of the hippocampus. Other brain regions with Aβ42 staining included subiculum **(G)**, inferior colliculus **(H)**, piriform cortex **(I)**, striatum **(J)**, motor cortex **(K)** and sensory cortex **(L)**. Scale bars: **(A)** = 200 μm; **(B,E)** = 100 μm; **(G,H,J–L)** = 50 μm; **(C,D,F,I)** = 33 μm.

**Figure 9:**
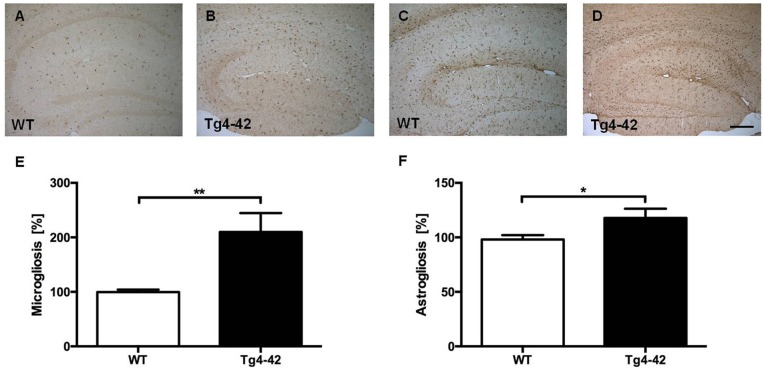
Increased microgliosis and astrogliosis in Tg4–42 mice.** (A,B,E)** Tg4–42 mice displayed significantly more reactive microglia activity in the brain compared to age-matched WT controls. **(C,D,F)** Tg4–42 mice exhibited ~20% more astroglia reactivity than WT controls. Scale bar: **(D)** = 200 μm. Unpaired *t*-test; **p < 0.01; *p < 0.05; data presented as mean ± SEM.

Brain sections of Tg4–42 mice were stained with an Aβ antibody to evaluate the expression of Aβ4–42 in the brain (Figure 8). Tg4–42 mice displayed strong intraneuronal Aβ-accumulation especially in the CA1 pyramidal cell layer (Figures 8A,E,F). Other exemplary shown brain regions with Aβ42 staining are DG, CA3 region of the hippocampus, subiculum, inferior colliculus, piriform cortex and striatum (Figures 8B–D, 8B–D, G–J). Furthermore the motor cortex and sensory cortex exhibited strong Aβ immunoreactivity (Figures 8K,L). In addition, aged Tg4–42 mice displayed a significantly increased microglia activity in the hippocampus (*t*-test: *p* = 0.0028; Figures 9A,B,E). Immunohistochemical GFAP staining revealed 20% more reactive astroglia in Tg4–42 mice compared to WT animals (*t*-test: *p* = 0.0390; Figures 9C,D,F).

## Discussion

In order to develop and evaluate new therapeutic approaches an early diagnosis of AD is crucial. Brain imaging displays a promising tool for a non-invasive early detection of AD. PET is an imaging technique that allows *in vivo* monitoring of pathological and physiological metabolic processes (Shukla and Kumar, 2006). In clinical routine, ^18^F-FDG-PET is an established method for differential diagnosis of clinically suspected indistinguishable dementia including AD. The target of ^18^F-FDG is the cerebral glucose metabolism which is mainly determined by synaptic activity. Neuronal dysfunction occurs long before neuron loss and can therefore function as an early marker of neurodegenerative diseases. The use of ^18^F-FDG-PET in order to assess dementias relies on the detection of cerebral areas with reduced glucose metabolism. Disease-specific patterns of lower ^18^F-FDG uptake help identifying different forms of dementia. Typical AD cases already show reduced ^18^F-FDG uptake within the posterior cingulate cortex in early stages (Minoshima et al., 1997; Matsuda, 2001; Jack et al., 2013). Impaired glucose metabolism then proceeds to the precuneus, posterior temporal and parietal cortex, often in a bilateral matter. In advanced stages of AD hypometabolism can also extend to the frontal lobe (Minoshima et al., 1997; Silverman et al., 2001; Foster et al., 2007; Teune et al., 2010). In preclinical research assessment of new therapeutic strategies in a non-invasive way is essential. Brain imaging with ^18^F-FDG-PET appears suitable for longitudinal therapy monitoring, showing neuron loss and neuronal dysfunction as major hallmarks of AD *in vivo*. However, earlier approaches using ^18^F-FDG-PET to study brain disorders in mice displayed some obstacles. One issue is the brain size relative to the resolution of the preclinical PET scanners as the volume of the mouse brain is about 3,000 times lower compared to humans (Welch et al., 2013). However, modern scanners and new reconstruction engines show a spatial resolution around 700 μm allowing detailed structural information of the brain with an exact distribution of the tracer. Another issue is the quantification of regional ^18^F-FDG uptake. With the use of a standardized mouse brain atlas VOI template and MRI co-registration this issue can also be resolved providing reliable and reproducible data.

A preclinical model for AD should display many features of the disease similar to the patient in order to achieve a successful translation into the clinic. Using ^18^F-FDG-PET for translational research of AD, a suitable mouse model should show similar changes in glucose metabolism as seen in AD patients. In the present study, Tg4–42 mice displayed changes in ^18^F-FDG-PET according to its AD pathologies. Aged Tg4–42 mice showed distinctly impaired glucose metabolism in all cortical areas correlating with the observed memory and neuron loss. While young Tg4–42 mice did not show significant differences in whole brain uptake compared to WT, glucose metabolism was reduced in the hippocampus, forebrain, hypothalamus, amygdala and midbrain. These regional changes in ^18^F-FDG uptake occurred even before memory deficits or neuron loss. We could previously show in electrophysiology experiments that young Tg4–42 mice display neuronal dysfunction and altered synaptic short-term plasticity (Dietrich et al., 2013). Our current *in vivo* results in the Tg4–42 model are also well in line with FDG-PET studies performed in AD patients where synaptic activity and glucose metabolism can be disturbed long before neuron loss and atrophy occur (Mosconi et al., 2010). The Tg4–42 mouse model shows changes in brain metabolism and synaptic dysfunction in an early disease stage similar to early changes observed in patients with AD.

Interestingly, cerebral metabolism seems to highly vary between different mouse models for AD. Different animal models of amyloidosis have shown unaltered, decreased as well as increased glucose metabolism (Kuntner et al., 2009; Luo et al., 2012; Li et al., 2016; Takkinen et al., 2017; Waldron et al., 2017). Cerebral glucose metabolism in AD mice has been previously studied in different AD mouse models using ^18^F-FDG or [^14^C]-2-deoxyglucose autoradiography. Similar to our finding several previous studies showed cerebral hypometabolism *ex-vivo* using autoradiography in different transgenic APP mouse models including PDAPP and the triple-transgenetic mouse model 3xTg (Valla et al., 2008; Nicholson et al., 2010). However, autoradiography requires euthanasia of the animals and therefore is not suitable for longitudinal studies or drug evaluations. The clinical phenotype of decreased ^18^F-FDG has also been reported *in vivo* in several AD models (Waldron et al., 2015, 2017). Notably, other AD mouse models did show no differences in brain metabolism or even cerebral hypermetabolism (Kuntner et al., 2009; Luo et al., 2012; Li et al., 2016; Takkinen et al., 2017). For example, an increased regional glucose metabolism was detected in the commonly used APP/PS1 mice at 2- and 12-months of age (Poisnel et al., 2012; Li et al., 2016). Furthermore, Rojas et al. (2013) reported increased ^18^F-FDG binding ratios relative to the cerebellum in aged 5XFAD mice. They hypothesized that the lack of glucose hypometabolism in many AD mouse models may be due to increased glia uptake of ^18^F-FDG. Amyloid plaques are pro-inflammatory agents and increased activated astroglia and microglia around them could lead to increased cerebral ^18^F-FDG uptake (Rojas et al., 2013; Waldron et al., 2017). However, AD patients display a decreased brain glucose metabolism despite the presence of amyloid plaques and increased gliosis (Herholz et al., 2007; Mosconi et al., 2010; Marcus et al., 2014). We could reaffirm this in the Tg4–42 model as mice also show increased inflammation and hypometabolism.

The lack of cerebral hypometabolism in many AD mouse models may also be due to the lack of neuron loss in most AD models. While atrophy and neuron loss are one of the major neuropathological hallmarks of AD, many transgenic AD mouse models lack this key feature (Bayer and Wirths, 2010; Schaeffer et al., 2011). Some of the earliest damages in AD brains have been described in the hippocampus. Padurariu et al. (2012) reported a decrease in neuronal density especially in the CA1 and CA3 regions of the hippocampus, although the effects in the CA1 area were more severe. The hippocampus is a brain area that is known to be highly affected in AD and contributes to the learning and memory deficits observed in AD patients. Therefore, we examined exemplary the neuron numbers in the hippocampus and extended previous findings demonstrating that Tg4–42 display a severe neuron loss not only in the CA1 region of the hippocampus but also in CA2 and CA3 region and the DG. The impaired spatial reference and recognition memory in aged Tg4–42 mice can be attributed to the neuronal disfunction, glucose metabolic impairments and the observed neuron loss.

Inconsistencies in previous ^18^F-FDG-PET AD animal studies might also be explained by different PET scanners, image acquisition and analysis methods. There is no standard for small animal brain imaging which results in high inter-study variations. Variants include tracer application, image acquisition and quantification methods. The effect of blood glucose on ^18^F-FDG uptake in the brain has also to be taken into consideration. Normalizing the SUV for blood glucose appears a suitable method to compensate for inequalities in glucose levels and fasting durations as cerebral ^18^F-FDG uptake shows an inverse relationship with blood glucose levels (Wong et al., 2011). Earlier studies demonstrated in repeated scans that brain ^18^F-FDG uptake values are well stabilized by glucose normalization (Wong et al., 2011; Deleye et al., 2016; Coleman et al., 2017).

It has to be noted while a wide range of animal models of AD have been generated and contributed to a better understanding of the pathogenesis of AD none of the models mimics the full range of pathological and degenerative alternations observed in AD patients (Götz and Ittner, 2008; Elder et al., 2010; Schaeffer et al., 2011; Webster et al., 2014). Most of these models only show certain aspects of the disease including amyloid plaques, neurofibrillary tangles, altered glucose metabolism, neuronal loss, gliosis or cognitive deficits. For example, the majority of transgenic mouse models expressing human APP show a robust plaque pathology but most of them lack a significant neuron loss (Duyckaerts et al., 2008; Cavanaugh et al., 2014). The Tg4–42 model used in this study shows key pathological features of AD including soluble amyloid Aβ oligomers, intraneuronal Aβ, glucose hypometabolism, neuron loss and memory deficits. However, it lacks neurofibrillary tangles and a robust plaque load pathology while showing region-specific intraneuronal Aβ deposits. Therefore, *in vivo* imaging with amyloid (or tau) PET is not applicable to this model. On a side note, growing evidence suggests that intraneuronal Aβ accumulation contributes to the pathological events in AD and is one of the first neurodegenerative alterations in the AD brain (Fernández-Vizarra et al., 2004). Grundke-Iqbal et al. (1989) already reported nearly 30 years ago that intracellular Aβ precedes Aβ plaque formation in AD patients. In addition, it has been shown that intracellular Aβ also appears before neurofibrillary tangles (Gouras et al., 2000; Fernández-Vizarra et al., 2004). Furthermore, it could be demonstrated that mainly Aβ42 variants accumulate within neurons in AD vulnerable regions (Aoki et al., 2008).

## Conclusion

The evaluation of new therapeutic strategies relies heavily on suitable animal models that mimic the pathological changes in AD. Here we could show that Tg4–42 mice display an age-dependent altered glucose metabolism accompanied with severe neuron loss and memory deficits. Similar to AD patients a significant regional decrease could be detected early in the disease progression in young Tg4–42 mice. The altered glucose metabolism coupled with age- and disease related cognitive decline of the Tg4–42 mouse model make it well-suited for utilization of preclinical testing of AD-relevant therapeutics. ^18^F-FDG-PET is a robust tool to display changes and monitor therapeutic effects *in vivo*. Repeated scans of the same animal allow a follow up during a long-term therapy improving accuracy by reducing the effect of inter-animal variances and reducing the total number of required animals. However, it is essential that animals are tested under standardized conditions with low variations between single animals and groups. High inter-study differences should be avoided in future studies in which a standard imaging protocol would be highly beneficial.

Overall, Tg4–42 could be a useful AD model to monitor the effects of various therapeutic strategies *in vivo* using ^18^F-FDG uptake as a therapeutic readout.

## Author Contributions

CB performed experiments, analyzed data, contributed to experimental design and wrote the manuscript. PH performed experiments and analyzed data. TF, CI, MS and NB performed experiments. CS and TB participated in the discussion of the results. YB conceived and designed the project, performed experiments, analyzed data and wrote the manuscript. All authors contributed to revising the manuscript and approved the final version.

## Conflict of Interest Statement

University Medicine Göttingen has been granted a patent on the Tg4–42 mouse model, on which TB is listed as an inventor. The remaining authors declare that the research was conducted in the absence of any commercial or financial relationships that could be construed as a potential conflict of interest.
